# Ghrelin does not impact the blunted counterregulatory response to recurrent hypoglycemia in mice

**DOI:** 10.3389/fendo.2023.1181856

**Published:** 2023-06-02

**Authors:** Kripa Shankar, Salil Varshney, Deepali Gupta, Bharath K. Mani, Sherri Osborne-Lawrence, Nathan P. Metzger, Corine P. Richard, Jeffrey M. Zigman

**Affiliations:** ^1^Center for Hypothalamic Research, Department of Internal Medicine, University of Texas Southwestern Medical Center, Dallas, TX, United States; ^2^Division of Endocrinology, Department of Internal Medicine, University of Texas Southwestern Medical Center, Dallas, TX, United States; ^3^Department of Psychiatry, University of Texas Southwestern Medical Center, Dallas, TX, United States

**Keywords:** ghrelin, hypoglycemia-associated autonomic failure, hypoglycemia unawareness, counterregulation, mouse models, Blood glucose

## Abstract

**Introduction:**

Recurrent episodes of insulin-induced hypoglycemia in patients with diabetes mellitus can result in hypoglycemia-associated autonomic failure (HAAF), which is characterized by a compromised response to hypoglycemia by counterregulatory hormones (counterregulatory response; CRR) and hypoglycemia unawareness. HAAF is a leading cause of morbidity in diabetes and often hinders optimal regulation of blood glucose levels. Yet, the molecular pathways underlying HAAF remain incompletely described. We previously reported that in mice, ghrelin is permissive for the usual CRR to insulin-induced hypoglycemia. Here, we tested the hypothesis that attenuated release of ghrelin both results from HAAF and contributes to HAAF.

**Methods:**

C57BL/6N mice, ghrelin-knockout (KO) + control mice, and GhIRKO (ghrelin cell-selective insulin receptor knockout) + control mice were randomized to one of three treatment groups: a “Euglycemia” group was injected with saline and remained euglycemic; a 1X hypoglycemia (“1X Hypo”) group underwent a single episode of insulin-induced hypoglycemia; a recurrent hypoglycemia (“Recurrent Hypo”) group underwent repeated episodes of insulin-induced hypoglycemia over five successive days.

**Results:**

Recurrent hypoglycemia exaggerated the reduction in blood glucose (by ~30%) and attenuated the elevations in plasma levels of the CRR hormones glucagon (by 64.5%) and epinephrine (by 52.9%) in C57BL/6N mice compared to a single hypoglycemic episode. Yet, plasma ghrelin was equivalently reduced in “1X Hypo” and “Recurrent Hypo” C57BL/6N mice. Ghrelin-KO mice exhibited neither exaggerated hypoglycemia in response to recurrent hypoglycemia, nor any additional attenuation in CRR hormone levels compared to wild-type littermates. Also, in response to recurrent hypoglycemia, GhIRKO mice exhibited nearly identical blood glucose and plasma CRR hormone levels as littermates with intact insulin receptor expression (floxed-IR mice), despite higher plasma ghrelin in GhIRKO mice.

**Conclusions:**

These data suggest that the usual reduction of plasma ghrelin due to insulin-induced hypoglycemia is unaltered by recurrent hypoglycemia and that ghrelin does not impact blood glucose or the blunted CRR hormone responses during recurrent hypoglycemia.

## Introduction

Insulin-induced hypoglycemia is a prevalent and serious issue affecting individuals with Type 1 diabetes mellitus and insulin-requiring Type 2 diabetes mellitus (1–3). In individuals without diabetes, hypoglycemia is usually countered by a highly integrated defense system which includes reduced insulin secretion, increased release of glucagon, glucocorticoids, epinephrine, norepinephrine, and GH, and stimulation of the sympathetic nervous system. Yet, in individuals with diabetes experiencing frequent hypoglycemia due to over-insulinization, this counterregulatory response (CRR) is often compromised. Specifically, as compared to individuals in whom an intact CRR initiates a constellation of sympathoadrenal warning symptoms (such as tachycardia, tremors, anxiety, irritability, arousal, sweating and hunger) that help prevent or correct hypoglycemia, individuals with diabetes exposed to repeated hypoglycemic episodes often lack these warning signals and fail to recognize hypoglycemia. This impaired CRR and the accompanying hypoglycemia unawareness comprise a condition known as hypoglycemia-associated autonomic failure (HAAF), and altogether further exacerbate the morbidity associated with hypoglycemia (4, 5).

The hormone ghrelin is well-positioned to serve a protective function during over-insulinization as a result of its many interactions with known mediators of blood glucose. For instance, ghrelin indirectly and directly interacts with pancreatic islet β-cells to reduce insulin secretion (6–9). Ghrelin also potentiates GH secretion and glucagon release, stimulates food intake, and raises circulating cortisol (9–13). Ghrelin’s overall glucoregulatory effects are emphasized by the actions of administered ghrelin to increase blood glucose (6, 9, 13–18) and conversely, by the blood glucose-lowering effects of GHSR blockade or ghrelin deletion, as reviewed in (10, 11). Pharmacological GHSR blockade improves hyperglycemia, glucose tolerance, and insulin sensitivity in diet-induced obese mice, Zucker diabetic fatty rats, and/or MODY-3 diabetic mice (19–21). Ghrelin-KO mice exhibit a progressive decline in fasting blood glucose to the point of near-death following a week-long caloric restriction regimen (daily access to 40% of usual calories) (22). Additionally, a functional ghrelin system protects against severe hypoglycemia in young mice subjected to acute fasting and prevents hypoglycemia in streptozotocin-treated mice lacking glucagon receptors (23).

Importantly, contributions of ghrelin to the insulin-induced hypoglycemia CRR also have been identified. Indeed, ghrelin-KO mice exhibit more pronounced and prolonged hypoglycemia than wild-type (WT) littermates when bolused i.p. with the same insulin dose (24). Both non-diabetic and streptozotocin-induced diabetic ghrelin-KO mice require 5.8-fold to 10-fold higher glucose infusion rates than WT littermates during low-dose hyperinsulinemic-hypoglycemic clamps (24, 25), similar to findings in GHSR-KO mice (26). Further, ghrelin-KO mice exhibit less robust corticosterone and GH responses (in the non-diabetic state) or less robust epinephrine and norepinephrine responses (in the diabetic state) than their WT counterparts during the clamps (24, 25). Collectively, these data suggest that endogenously-produced ghrelin not only influences insulin sensitivity, but also is permissive for the normal CRR to insulin-induced hypoglycemia (24, 25).

Ghrelin also is well-positioned to serve a protective function during over-insulinization as a result of the ability of ghrelin cells to directly sense glucose, insulin, and sympathetic nervous system activity. Specifically, although evidence for a direct effect of glucose on ghrelin cells *in vivo* is not available, ghrelin secretion from cultured gastric mucosal cells is increased in low glucose conditions and inhibited by high glucose (27). Insulin acts directly on ghrelin cell-expressed insulin receptors to restrict ghrelin release – a phenomenon which has been demonstrated *in vivo* using GhIRKO (ghrelin cell-selective insulin receptor knockout) mice (28). An effect of insulin to suppress ghrelin release also has been shown in human studies. For instance, one clinical trial showed that plasma ghrelin falls both following a short insulin infusion leading to hypoglycemia and following a short insulin infusion plus dextrose infusion to maintain euglycemia (29). Another clinical trial showed a fall in ghrelin during a hyperinsulinemic-euglycemic clamp procedure (30). Yet a third clinical trial showed that hypoglycemia resulting from a single i.v. insulin injection initially lowers ghrelin and then leads to a ghrelin rebound 1 h later (31). In contrast to the effect of insulin, norepinephrine released from stomach-projecting sympathetic neurons acts directly on ghrelin cell-expressed β_1_-adrenergic receptors to stimulate ghrelin release (27, 32–38). Ghrelin release increases when sympathetic nerves are stimulated artificially or when adrenergic agents are infused into the gastric mucosa (35, 38). Norepinephrine and epinephrine both potently stimulate ghrelin secretion from ghrelinoma cell lines and 1° cultures of dispersed gastric mucosal cells (27, 32–37). Also, ghrelin cell-selective deletion of β_1_-adrenergic receptors markedly blunts the usual calorie restriction-induced stimulation of ghrelin secretion, causing frank hypoglycemia in the above-described week-long caloric restriction regimen (39). Thus, each of these three inputs to the ghrelin cell – namely, glucose, insulin, and sympathetic nervous system activity – likely plays a balanced part in determining the amount of ghrelin released upon insulin-induced hypoglycemia.

In the setting of insulin-induced recurrent hypoglycemia resulting in HAAF, we hypothesize that the failed autonomic response, which presumably includes blunted exposure of ghrelin cells to norepinephrine released from stomach-projecting sympathetic neurons, might result in even lower ghrelin levels than usual following an occasional episode of insulin-induced hypoglycemia. In turn, because of ghrelin’s capacity to engage multiple arms of the CRR, we predict that this impaired ghrelin response contributes to the attenuated CRR that characterizes HAAF. To test this hypothesis, we have established a recurrent hypoglycemia mouse model, which has been adapted from the literature. We submitted C57BL/6N mice, ghrelin-KO mice, and GhIRKO mice to recurrent hypoglycemia and two control protocols and then compared blood glucose, plasma ghrelin levels, and plasma levels of the CRR hormones glucagon, epinephrine, and norepinephrine.

## Methods

### Animals

**Figure 1:**
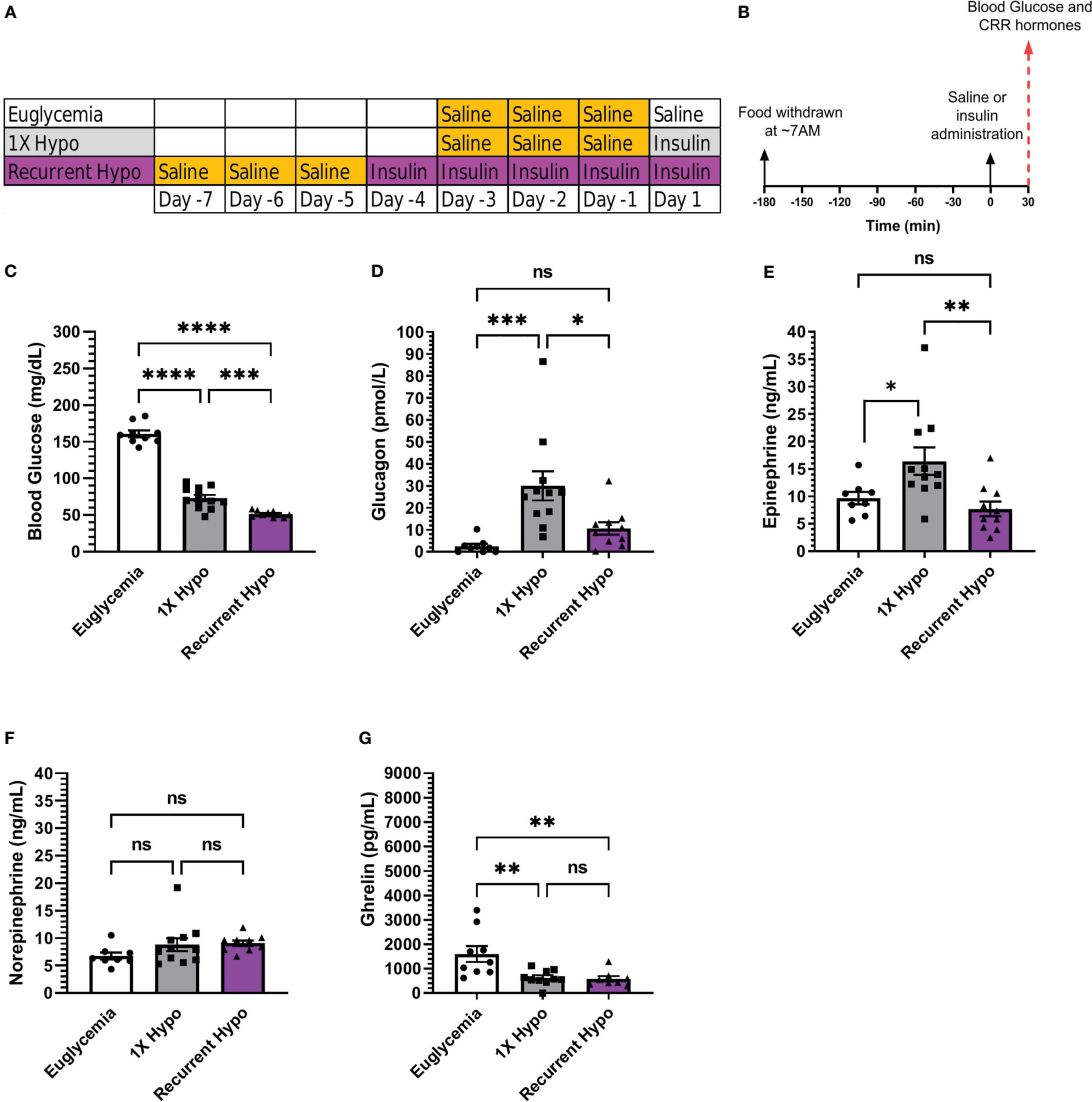
Validation of a recurrent hypoglycemia model of hypoglycemia-associated autonomic failure in C57BL/6N mice. **(A)** Table outlining the days on which mice in the “Euglycemia”, “1X Hypo”, and “Recurrent Hypo” groups were acclimated to i.p. injections (via handling and saline injections; highlighted in gold) and were administered saline on the final Day (“Euglycemia”), insulin on the final day (“1X Hypo”), or insulin for 5 days in a row (“Recurrent Hypo”). **(B)** Schematic diagram depicting the schedule on the days of antecedent hypoglycemia and the final Days. **(C)** Blood glucose levels, **(D)** plasma glucagon, **(E)** plasma epinephrine, **(F)** plasma norepinephrine, and **(G)** plasma ghrelin measured on the final day of the “Euglycemia”, “1X Hypo” and “Recurrent Hypo” protocols at 30 min post i.p. injection of saline or insulin. Data are represented as mean ± SEM and were analyzed by one-way ANOVA followed by Tukey’s multiple comparison test. n=9-12. Statistically significant differences are indicated by asterisks: *P < 0.05, **P < 0.01, ***P < 0.001, and ****P < 0.0001. ns represents no statistical significance.

**Figure 2:**
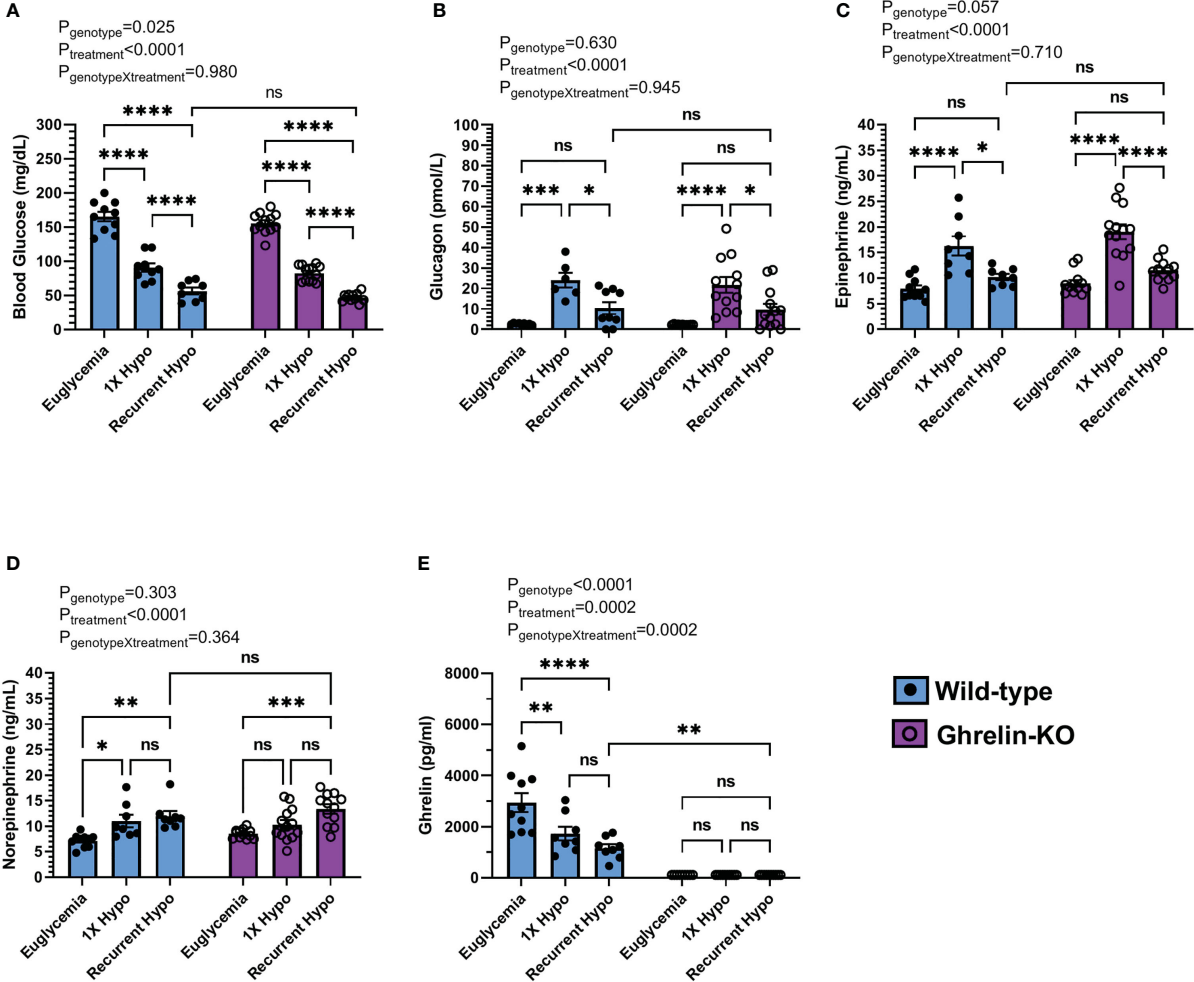
Effect of ghrelin deletion on blood glucose and the CRR following recurrent hypoglycemia. **(A)** Blood glucose levels, **(B)** plasma glucagon, **(C)** plasma epinephrine, **(D)** plasma norepinephrine, and **(E)** plasma ghrelin at 30 min post i.p injection of saline or insulin on the final day of the “Euglycemia”, “1X Hypo”, and “Recurrent Hypo” protocols. Data are represented as mean ± SEM and were analyzed by two-way ANOVA followed by Sidak’s multiple comparison test. n=8-13. Effects of genotype, treatment, and the interaction between the two are listed above the panels whereas post-hoc analysis results appear as connecting lines and asterisks above the bars. Statistically significant differences are indicated by asterisks: *P < 0.05, **P < 0.01, ***P < 0.001, and ****P < 0.0001. ns represents no statistical significance.

**Figure 3:**
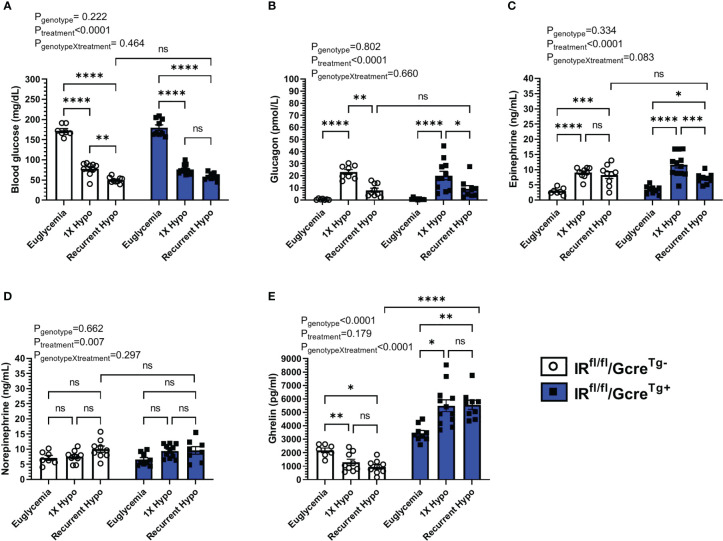
Effect of raising plasma ghrelin as a result of ghrelin cell-selective IR deletion on blood glucose and the CRR to recurrent hypoglycemia. **(A)** Blood glucose levels, **(B)** plasma glucagon, **(C)** plasma epinephrine, **(D)** plasma norepinephrine, and **(E)** plasma ghrelin at 30 min post i.p injection of saline or insulin on the final day of the “Euglycemia”, “1X Hypo”, and “Recurrent Hypo” protocols. Data are represented as mean ± SEM and were analyzed by two-way ANOVA followed by Sidak’s multiple comparison test. n=9-12. Effects of genotype, treatment, and the interaction between the two are listed above the panels whereas post-hoc analysis results appear as connecting lines and asterisks above the bars. Statistically significant differences are indicated by asterisks: *P < 0.05, **P < 0.01, ***P < 0.001, and ****P < 0.0001. ns represents no statistical significance.

Male C57BL/6N mice (8 – 10 weeks-of-age) were used for **Figure 1**. Male ghrelin-KO and WT littermates on a C57BL/6N background (8 – 10 weeks-of-age), which were generated by pairing mice heterozygous for the ghrelin-KO allele, were used for **Figure 2**. The ghrelin-KO line (GKO1) used here was derived in (40) and validated by demonstrating absent stomach ghrelin-immunoreactivity and undetectable plasma ghrelin (24). GhIRKO and floxed-insulin receptor (floxed-IR) littermates on a C57BL/6N background (8 – 10 weeks-of-age), which were generated by crossing a validated Ghrelin-cre mouse line (37, 39) with the Kahn lab’s conditional IR-KO (IR^fl/fl^; floxed-IR; B6.129S4 [FVB]-Insrtm1Khn/J; The Jackson Laboratory) mouse line (41), as described in (28), were used for **Figure 3**. GhIRKO (IR^fl/fl^/Gcre^Tg+^) mice contain two copies of the floxed-IR gene and one copy of the Ghrelin-cre transgene; control floxed-IR (IR^fl/fl^/Gcre^Tg–^) littermates contain two copies of the floxed-IR gene and no copies of the Ghrelin-cre transgene. Mice were housed at 21.5–22.5°C using a 12-h light-dark cycle and were provided *ad libitum* access to water and regular chow (2016 Teklad Global 16% Protein Rodent Diet; Envigo, Indianapolis, IN), except as indicated. Experiments were approved by the UT Southwestern Medical Center Institutional Animal Care and Use Committee.

### Recurrent hypoglycemia mouse model

To establish a mouse model of recurrent hypoglycemia, we modified a HAAF protocol reported by the C. Mobbs lab (42). Mice randomized to the recurrent hypoglycemia (“Recurrent Hypo”) group were singly housed for 7 days. Over the next 3 days, while remaining singly housed, each mouse was handled and injected with saline (5 µL/g body weight) to acclimatize them. Over the next 4 days (while still singly housed), food was withdrawn at ~7:00 AM (just after lights-on); 3 hr later, mice were subjected to a daily hypoglycemic episode induced by administering a single i.p. bolus of Humulin-R insulin 2.5 units/kg body weight (Eli Lilly, Indianapolis, IN); blood glucose was measured from nicked tails using a Bayer Contour Next EZ blood glucose monitoring system (Ascensia Diabetes Care, Parsippany, NJ) at 0, 30, 90, 120, and 240 min following insulin administration; for the ghrelin-KO + WT littermate mice, 100 μL of a 10% dextrose solution was administered by oral gavage to those mice in which a blood glucose < 35 mg/dL was recorded (notably, throughout the 4-day acclimation period, blood glucose levels < 35 mg/dL were not noted until the 90 min timepoint, occurred only on the 1^st^ day of insulin administration, and occurred only in 6 of 12 ghrelin-KO mice and 1 of 8 WT littermates); *ad lib* access to food was again provided at 240 min following insulin administration. On the 5^th^ day, the above protocol was repeated for the mice in the “Recurrent Hypo” group, except that at 30 min following insulin administration, mice were sacrificed by quick decapitation.

Mice randomized to a “Euglycemia” control group underwent 11 days of single housing and 3 days of handling + saline injections while remaining singly housed (as described above). The following day (while still singly housed), food was withdrawn at ~7:00 AM (just after lights-on); 3 hr later, mice were administered a single i.p. bolus of saline (5 µL/g body weight); blood glucose was measured from nicked tails, as above at 0 and 30 min following saline administration; mice were sacrificed by quick decapitation at 30 min following saline administration. Mice randomized to a 1X hypoglycemia (“1X Hypo”) control group underwent the same protocol as the “Euglycemia” control group except instead of receiving a single i.p. bolus of saline on the day that food was withdrawn, they were administered a single i.p. bolus of Humulin-R insulin 2.5 units/kg body weight. A Table and schematic diagram depicting these protocols can be found in **Figures 1A, B**.

Of note, the insulin dose used produced hypoglycemia without producing unconsciousness, seizures, or mortality. For the first 4 days of insulin administration, mice in the “Recurrent Hypo” developed hypoglycemia (mean blood glucose = 62 ± 3 mg/dL) by 30 min following insulin administration, remained hypoglycemic (mean blood glucose = 59 ± 2 mg/dL) until at least 120 min following insulin administration, and became euglycemic (mean blood glucose = 109 ± 7 mg/dL) by 240 minutes after insulin administration. For all groups, trunk blood to assess plasma levels of ghrelin (the acylated form), glucagon, corticosterone, epinephrine, and norepinephrine was collected into ice-cold 3 mL EDTA-coated vacutainer tubes (BD Biosciences, Franklin Lakes, NJ) at the time of sacrifice.

Modifications to the “Recurrent Hypo” and control protocols previously reported in (42) included background genetic strain [C57BL/6J in (42)], age of mice [12 weeks-of-age in (42)], acclimatization protocol [1X Hypo and Euglycemia groups: 4 days of saline injections during which food was withdrawn and blood glucose measurements were made in (42); Recurrent hypo group: no saline acclimatization in (42)], timing of blood glucose determinations on the final day [0, 30, 90, 180, and 240 min in (42)], duration of food restriction during days of antecedent hypoglycemia [180 min in (42)], timing of CRR hormone measurements [4 hrs after the 5th insulin injection in (42)], use of anesthesia [brief exposure to carbon dioxide was used in (42) just prior to decapitation and collection of trunk blood], and use of dextrose administration to correct blood glucose < 35 mg/dL [not used in (42)].

### Determination of plasma hormone levels

For ghrelin, blood was aliquoted into ice-cold microtubes. P-hydroxymercuribenzoic acid (final concentration 1 mM) (Sigma-Aldrich, St. Louis, MO) was added, plasma was isolated following centrifugation, and HCl was added to achieve a final concentration of 0.1 N. For other hormones, blood was collected into three different microtubes. For glucagon, aprotinin (final concentration 250 KIU/mL) (Sigma-Aldrich) was added. For catecholamines, EDTA-glutathione solution (9% w/v EDTA and 6% w/v glutathione, pH 7.4; 2 μL per 100 μL blood) was added. ELISA kits were used for acyl-ghrelin (Millipore-Merck, Burlington, MA) and glucagon (Mercodia AB, Uppsala, Sweden). Calorimetric assays were performed using a BioTek PowerWave XS Microplate spectrophotometer (BioTek, Winooski, VT) and BioTek KC4 junior software. Plasma catecholamines were determined using high-performance liquid chromatography at the Vanderbilt University Medical Center Hormone Assay and Analytical Services Core.

### Statistical analyses

Data are represented as mean ± SEM. One-way ANOVA followed by Tukey’s multiple comparisons test or two-way ANOVA followed by Sidak’s comparisons test was used to test for significant differences among test groups. Data with significant unequal variance were log transformed prior to performing analyses. Outliers, if any were detected by the ROUT (robust regression and outlier removal) test. Statistical significance was defined as *P* < 0.05. All statistical analyses and graph preparations were performed using GraphPad Prism 9.3.1.

## Results

### Recurrent hypoglycemia exaggerates the fall in blood glucose and impairs the CRR in C57BL/6N mice

Humulin R (2.5 U/Kg) was bolused i.p. to transiently induce hypoglycemia in male C57BL/6N mice. Insulin administration lowered blood glucose by 30 min to 73 ± 4 mg/dL in “1X Hypo” mice whereas control “Euglycemia” mice had blood glucoses of 161 ± 5 mg/dL 30 min following saline administration. Mice in the “Recurrent Hypo” group, which experienced five consecutive days of hypoglycemic episodes, exhibited a mean blood glucose (51 ± 1 mg/dL) 30 min following insulin administration that was significantly lower (by ~30%) than that exhibited by the “1X Hypo” group (**Figure 1C**). Also, the rise in plasma levels of two CRR hormones normally observed following a single episode of hypoglycemia (30 min following insulin administration) was significantly attenuated in the “Recurrent Hypo” mice (**Figures 1D, E**). Notably, glucagon was lower by ~64.5% and epinephrine was lower by ~52.9% in “Recurrent Hypo” mice vs. “1X Hypo” mice (**Figures 1D, E**). No statistically significant differences between glucagon or epinephrine levels were observed in “Recurrent Hypo” mice vs. “Euglycemia” mice (**Figures 1D, E**). Neither 1X hypoglycemia nor recurrent hypoglycemia significantly altered the plasma norepinephrine levels measured at the 30 min timepoint (**Figure 1F**). Further, insulin treatment significantly suppressed plasma ghrelin, although no significant differences were apparent between “Recurrent Hypo” mice and “1X Hypo” mice (**Figure 1G**). To summarize, five consecutive days of insulin-induced hypoglycemic episodes significantly lowered blood glucose and markedly blunted the rise in the CRR hormones glucagon and epinephrine, as expected for HAAF.

### Ghrelin deletion does not further impair the CRR following recurrent hypoglycemia

Next, we investigated the effect of ghrelin deletion on blood glucose and the CRR following recurrent hypoglycemia by comparing ghrelin-KO mice to WT littermates. In WT littermates, recurrent hypoglycemia resulted in a mean blood glucose (56 ± 5 mg/dL) 30 min following insulin administration that was lower (by ~37.9%) than that exhibited by the “1X Hypo” group (**Figure 2A**); the rise in plasma glucagon and epinephrine observed in the “1X Hypo” group was attenuated in the “Recurrent Hypo” group (**Figures 2B, C**); plasma ghrelin was lowered by both the “1X Hypo” and “Recurrent Hypo” protocols to a similar degree (**Figure 2E**). These observations were similar to those made for C57BL/6N mice (**Figures 1C–E, G**). Unlike that observed in C57BL/6N mice (**Figure 1F**), norepinephrine was raised by both the “1X Hypo” and “Recurrent Hypo” protocols in the WT littermates (**Figure 2D**).

No significant interactions between genotype and treatment were observed for blood glucose, plasma glucagon, epinephrine, or norepinephrine (**Figures 2A–D**). In other words, the patterns of changes induced by the “1X Hypo” and “Recurrent Hypo” protocols present in WT littermates were similar for ghrelin-KO mice. Ghrelin-KO mice exhibited neither exaggerated hypoglycemia in response to recurrent hypoglycemia, nor any additional attenuation in glucagon or epinephrine compared to WT littermates.

### Increasing ghrelin *via* ghrelin cell-selective insulin receptor deletion does not augment the CRR following recurrent hypoglycemia

Finally, we took advantage of GhIRKO mice to investigate the effect of raising ghrelin on blood glucose and the CRR following recurrent hypoglycemia. Previously, we used these mice to demonstrate that ghrelin cell-expressed insulin receptors are required for insulin-mediated reductions in plasma ghrelin (28). Specifically, while insulin lowered plasma ghrelin in floxed-IR control mice, plasma ghrelin rose from baseline during insulin infusion in GhIRKO mice (28). Additionally, GhIRKO mice required reduced glucose infusion rates during hyperinsulinemic-hypoglycemic clamps, suggesting that suppressed ghrelin release resulting from direct insulin action on ghrelin cells usually limits ghrelin’s full potential to protect against insulin-induced hypoglycemia (28).

In floxed-IR control mice, recurrent hypoglycemia resulted in a mean blood glucose (50 ± 2 mg/dL) 30 min following insulin administration that was lower (by ~35%) than that exhibited by the “1X Hypo” group (**Figure 3A**); the rise in plasma levels of glucagon observed in the “1X Hypo” group was attenuated in the “Recurrent Hypo” group (**Figure 3B**); plasma ghrelin was lowered by both the “1X Hypo” and “Recurrent Hypo” protocols to a similar degree (**Figure 3E**). These observations were similar to what had been observed for C57BL/6N mice (**Figure 1**) and for the WT littermates from the ghrelin-KO study (**Figure 2**).

Just as had been observed for the ghrelin-KO and WT cohort, no significant interactions between genotype (GhIRKO mice vs. control floxed-IR littermates) and treatment were observed for blood glucose, plasma glucagon, epinephrine, or norepinephrine (**Figures 3A–D**). In other words, the patterns of changes induced by the “1X Hypo” and “Recurrent Hypo” protocols present in floxed-IR mice were similar for GhIRKO mice, despite significantly higher plasma ghrelin in GhIRKO mice (**Figure 3E**).

## Discussion

In the current study, we established a recurrent hypoglycemia protocol to model HAAF in mice and to determine if alterations in plasma ghrelin occur in HAAF and contribute to the attenuated CRR that characterizes HAAF. We successfully adapted a published HAAF model, demonstrating that 4 antecedent episodes of insulin-induced hypoglycemia resulted in a greater drop in blood glucose and markedly attenuated glucagon and epinephrine CRRs when assessed after a 5^th^ episode of insulin-induced hypoglycemia as compared to controls that experienced only a single insulin-induced hypoglycemic episode. Yet, plasma ghrelin was equivalently suppressed in “1X Hypo” and “Recurrent Hypo” C57BL/6N mice. Also, no statistically significant genotype X treatment effects were observed in the ghrelin-KO + WT littermate cohorts submitted to the “Euglycemia”, “1X Hypo”, or “Recurrent Hypo” protocols or in the GhIRKO + floxed-IR littermate cohorts submitted to the “Euglycemia”, “1X Hypo”, or “Recurrent Hypo” protocols. This suggests that neither ghrelin-KO mice, which lack ghrelin, nor GhIRKO mice, which have high ghrelin due to the inability of insulin to suppress ghrelin release, responded differently to the treatments than their controls. In other words, reducing ghrelin or increasing ghrelin did not impact the aberrant CRR induced by recurrent hypoglycemia.

One notable finding worthy of discussion relates to the precision of the recurrent hypoglycemia + associated control protocols used here as a model for HAAF. Modeling HAAF to understand it mechanistically has been a challenge in both human and animal studies (5, 43), but perhaps more-so in animal studies, particularly mice. Whereas rats are mostly used, transgenic modifications that aid mechanistic studies are more routine in mice, which is a main reason why mice were chosen for the current study. In our efforts to establish a working model that allowed the incorporation of mouse genetics to investigate the contribution of ghrelin to HAAF, we sought the guidance of published mouse HAAF models. Using the search terms “hypoglycemia-associated autonomic failure” OR “hypoglycemia unawareness” OR “recurrent hypoglycemia” AND “mouse”, we originally found 9 publications on the https://pubmed.ncbi.nlm.nih.gov website (42, 44–51). All used different recurrent hypoglycemia protocols and only some demonstrated features of HAAF – specifically, more profound and/or prolonged hypoglycemia plus attenuated rises in CRR hormone levels (42, 44–51). Only one study included diabetic mice and only 4 used transgenic mouse models (44, 47, 48, 51). Our challenges were to optimize details of the recurrent hypoglycemia protocol (including duration and severity of hypoglycemic episodes, number of episodes, spacing of episodes, type of insulin, administration route, use of insulin vs. the glucoprivic agent 2-deoxy-D-glucose, and amount of fasting), to design appropriate accompanying control protocols, and to determine how best to objectively measure HAAF biochemically (for instance, whether to measure blood glucose alone or together with CRR hormones while also considering the timing and site of the blood draws) or otherwise (particularly, whether to consider any specific physiological and/or behavioral measures of a failed sympathoadrenal response).

As mentioned, the protocol we present here is adapted from the Charles Mobbs lab (42), with the modifications made to that protocol noted in the Methods section. Not only do we reproduce the finding by the Mobbs lab of more profound hypoglycemia (at 30 min) for the “Recurrent Hypo” group (vs. the “1X Hypo” group), but also we describe attenuated rises of CRR hormones – here, glucagon and epinephrine at 30 min after the 5^th^ insulin injection vs. glucagon and corticosterone at 4 hrs after the 5th insulin injection in (42). Thus, we successfully have emulated several of the biochemical findings characteristic of HAAF. Notably, our protocol did not include any behavioral tests that mimic the usual sympathoadrenal warning symptoms that are lost in HAAF. For instance, we did not observe differential degrees of hypoglycemia-induced hyperphagia between “Recurrent Hypo” and “1X Hypo” C57BL/6N groups (data not shown) as has been observed in rats (52). Nor did we incorporate any behavioral surrogates for hypoglycemia unawareness such as loss of conditioned place preference for food rewards, as was recently reported in another mouse HAAF model (53) that was not available when we began our studies.

It also is worthwhile to discuss potential reasons why the ghrelin response to over-insulinization was not altered by recurrent hypoglycemia. As mentioned in the Introduction, insulin and sympathetic nervous system-derived norepinephrine both exert substantive direct effects on ghrelin secretion. Insulin engages ghrelin cell-expressed insulin receptors to restrict ghrelin release whereas norepinephrine from the sympathetic nervous system engages ghrelin cell-expressed β_1_-adrenergic receptors to stimulate ghrelin release. Activating ghrelin cell-expressed β_1_-adrenergic receptors helps maintain basal plasma ghrelin and mediates caloric restriction-induced rises in plasma ghrelin and probably also stress-induced rises in plasma ghrelin (39, 54). Thus, one might assume that the attenuated sympathetic nervous system response associated with HAAF (e.g. the autonomic failure part of HAAF) would result in even less ghrelin release than usual as a consequence of an occasional episode of over-insulinization. Yet, plasma ghrelin in the “Recurrent Hypo” was equivalent to that in the “1X Hypo” groups in both the C57BL/6N mice and in the WT littermates of the ghrelin-KO mice. The occurrence of similar plasma ghrelin in the “Recurrent Hypo” and “1X Hypo” groups could result from the observed plasma norepinephrine levels being not only equivalent in those groups, but also rather high (in the ng/mL range compared with the pg/mL range, most likely as a result of the methods used for blood collection) in both groups.

It also is possible that the exaggerated fall in blood glucose in the “Recurrent Hypo” group might have directly influenced ghrelin secretion. To expand on this idea further, whereas the inhibitory effects on ghrelin release by insulin and the stimulatory effects on ghrelin release by the sympathetic nervous system are predicted to be off-balance in recurrent hypoglycemia vs. 1X hypoglycemia, this may be offset by the more pronounced hypoglycemia in the setting of recurrent hypoglycemia. Specifically, the exaggerated hypoglycemia of “Recurrent Hypo” mice vs. “1X Hypo” mice might directly act on ghrelin cells to stimulate greater ghrelin secretion, thus compensating for any reduced ghrelin secretion resulting from an attenuated sympathetic nervous system response. Indeed, culturing gastric mucosal cells in media with 1 mM glucose (representative of hypoglycemia) results in much greater ghrelin release than from media with 5 mM glucose (representative of euglycemia) or 10 mM glucose (representative of hyperglycemia) (55, 56). Lowering glucose from 1 mM to 0 mM further enhances ghrelin release from cultured gastric mucosal cells (56). As mentioned above, while these *ex vivo* findings of a direct effect of low glucose on ghrelin release from ghrelin cells has not yet been confirmed *in vivo*, findings such as those in **Figure 3E** of elevated plasma ghrelin in hypoglycemic vs. euglycemic GhIRKO mice support this hypothesis.

It also remains unclear why genetic manipulation of ghrelin levels – as achieved in ghrelin-KO mice and in GhIRKO mice – did not modulate the CRR in the setting of recurrent hypoglycemia. This is in contrast to the demonstrated permissive effect of ghrelin on the CRR in the setting of 1X hypoglycemia. In particular, as compared to WT littermates, ghrelin-KO mice experience greater hypoglycemia following a 1X i.p. bolus of Humulin-R insulin (2.5 U/kg; noticeable at 90 min to at least 240 min following the insulin bolus, but not at 30 min or 60 min) (24). Also, here we showed that insulin reduced blood glucose to < 35 mg/dL in half of ghrelin-KO mice whereas it had the same effect in only one-eighth of WT littermates. Furthermore, both diabetic and non-diabetic ghrelin-KO mice require greater glucose infusion rates and exhibit attenuated CRR responses during a low-dose hyperinsulinemic-hypoglycemic clamp, as compared to WT littermates (24, 25). In contrast, as compared to floxed-IR littermates, GhIRKO mice require a lower glucose infusion rate and exhibit an exaggerated GH CRR during a low-dose hyperinsulinemic-hypoglycemic clamp procedure (28). The lack of either a more attenuated CRR to recurrent hypoglycemia in ghrelin-KO vs. WT littermates or a less attenuated CRR to recurrent hypoglycemia in GhIRKO vs. floxed-IR littermates suggests that ghrelin is not essential in regulating the CRR induced by recurrent hypoglycemia as it is in the settings of a one-time episode of hypoglycemia or a one-time hyperinsulinemic-hypoglycemic clamp protocol. We can only speculate that recurrent hypoglycemia changes sensitivity to ghrelin such that its effects on blood glucose and CRR hormones identified in single episode hypoglycemia paradigms can no longer be engaged.

## Data availability statement

The raw data supporting the conclusions of this article will be made available by the authors, without undue reservation.

## Ethics statement

The animal study was reviewed and approved by UT Southwestern Medical Center Institutional Animal Care and Use Committee.

## Author contributions

KS conceptualized and performed the experiments, analyzed and interpreted the data, and helped write the manuscript. SV, DG, BM, and SO-L performed the experiments helped analyze and interpret the data. NM and CR maintained the animal colony and helped in some experiments. JZ conceptualized the experiments, secured funding, interpreted the data, supervised the research activity, and helped write the manuscript. All authors contributed to the article and approved the submitted version.
